# Osteoporosis Due to Hormone Imbalance: An Overview of the Effects of Estrogen Deficiency and Glucocorticoid Overuse on Bone Turnover

**DOI:** 10.3390/ijms23031376

**Published:** 2022-01-25

**Authors:** Chu-Han Cheng, Li-Ru Chen, Kuo-Hu Chen

**Affiliations:** 1 Department of Physical Medicine and Rehabilitation, Mackay Memorial Hospital, Taipei 104, Taiwan; chelsea830512@gmail.com (C.-H.C.); gracealex168@gmail.com (L.-R.C.); 2 Department of Mechanical Engineering, National Yang Ming Chiao Tung University, Hsinchu 300, Taiwan; 3 Department of Obstetrics and Gynecology, Taipei Tzu-Chi Hospital, The Buddhist Tzu-Chi Medical Foundation, Taipei 231, Taiwan; 4 School of Medicine, Tzu-Chi University, Hualien 970, Taiwan

**Keywords:** osteoporosis, estrogen, glucocortcoid

## Abstract

Osteoporosis is a serious health issue among aging postmenopausal women. The majority of postmenopausal women with osteoporosis have bone loss related to estrogen deficiency. The rapid bone loss results from an increase in bone turnover with an imbalance between bone resorption and bone formation. Osteoporosis can also result from excessive glucocorticoid usage, which induces bone demineralization with significant changes of spatial heterogeneities of bone at microscale, indicating potential risk of fracture. This review is a summary of current literature about the molecular mechanisms of actions, the risk factors, and treatment of estrogen deficiency related osteoporosis (EDOP) and glucocorticoid induced osteoporosis (GIOP). Estrogen binds with estrogen receptor to promote the expression of osteoprotegerin (OPG), and to suppress the action of nuclear factor-κβ ligand (RANKL), thus inhibiting osteoclast formation and bone resorptive activity. It can also activate Wnt/β-catenin signaling to increase osteogenesis, and upregulate BMP signaling to promote mesenchymal stem cell differentiation from pre-osteoblasts to osteoblasts, rather than adipocytes. The lack of estrogen will alter the expression of estrogen target genes, increasing the secretion of IL-1, IL-6, and tumor necrosis factor (TNF). On the other hand, excessive glucocorticoids interfere the canonical BMP pathway and inhibit Wnt protein production, causing mesenchymal progenitor cells to differentiate toward adipocytes rather than osteoblasts. It can also increase RANKL/OPG ratio to promote bone resorption by enhancing the maturation and activation of osteoclast. Moreover, excess glucocorticoids are associated with osteoblast and osteocyte apoptosis, resulting in declined bone formation. The main focuses of treatment for EDOP and GIOP are somewhat different. Avoiding excessive glucocorticoid use is mandatory in patients with GIOP. In contrast, appropriate estrogen supplement is deemed the primary treatment for females with EDOP of various causes. Other pharmacological treatments include bisphosphonate, teriparatide, and RANKL inhibitors. Nevertheless, more detailed actions of EDOP and GIOP along with the safety and effectiveness of medications for treating osteoporosis warrant further investigation.

## 1. Introduction

Osteoporosis is a serious health issue among aging postmenopausal females, who are at an increased risk of fracture. Bone mass is increased in childhood, reaching a peak level by the woman’s third or fourth decade of life. Thereafter, bone loss begins and accelerates at menopause. One study has reported that the rate of osteoporosis approximately doubled every 5 years, starting from the age of 45–49 with 3.3%, and progressively increasing to 50.3% at the age of 85 years and older [1].

The majority of postmenopausal females with osteoporosis have bone loss related to estrogen deficiency. The rapid bone loss results from an increase in bone turnover with an imbalance between bone resorption and bone formation.

Glucocorticoids are the most widely used anti-inflammatory drug around the world. Despite their excellent effect in managing many acute inflammatory diseases and autoimmune disorders, the usage of glucocorticoids has been limited due to substantial adverse effects. One of the most well-known side effects is osteoporosis, which further induces bone fracture and other musculoskeletal problems. In glucocorticoid-induced osteoporosis (GIOP), glucocorticoids induce bone demineralization with significant changes of spatial heterogeneities of bone at microscale, indicating potential risk of fracture [2,3]. It is estimated that the prevalence of GIOP in the general population ranges from 0.5% to 1% with a dose-dependent effect [4]. The highest bone loss rate occurs in the first 6 months, with additional risk factors associated with primary diseases, such as chronic liver disease, chronic kidney disease, inflammatory bowel disease, autoimmune disorder, and COPD [5]. Due to changes in bone metabolism, these co-morbidities cause additional bone loss relative to glucocorticoids use alone. The impact of underlying diseases has also been recognized in children [6]. Cumulative steroid dosage, older age, female sex, and low body weight are also major risk factors of metabolic fractures [7].

GIOP has drawn public concerns to become an important health issue, and several recommendations of prevention and treatment have been published, including the American College of Rheumatology’s (ACR) guideline, which was updated in 2001 and 2010, with the latest version in 2017 [8,9,10]. Despite increasing information about GIOP, it remains clinically underdiagnosed and undertreated [11,12,13], raising concerns that enhancing education among physicians are needed [14,15,16]. Even among long-term steroid users, such as rheumatoid arthritis patients, less than 40% received recommended DEXA scanning and treatment [17,18]. In addition to discussions regarding the mechanisms, actions, and effects of glucocorticoids on bone, assessment and management of GIOP will be included as well. The understanding of the mechanisms of hormone imbalance underlying estrogen deficiency related osteoporosis (EDOP) and GIOP is essential for future prevention and treatment. This review aimed to explore the molecular mechanisms of actions, the risk factors, and treatment of patients with hormone-related osteoporosis, including EDOP and GIOP.

## 2. Searching Terms and Strategies for the Literature 

**Figure 1:**
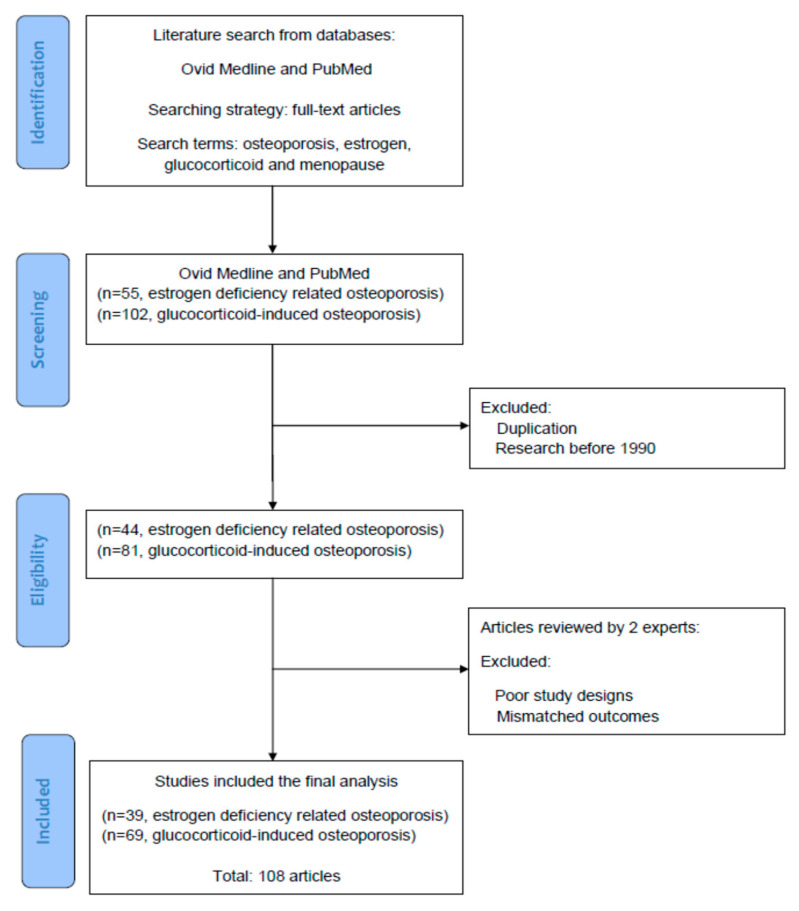
The diagram of article selection, screening, and inclusion from the literature.

Figure 1 illustrates the screening, selection, and inclusion of the references we retrieved in the literature. In the current review, all of the reference articles were solicited from the databases Medline and PubMed using the search terms “osteoporosis”, “estrogen”, “glucocorticoid”, and “menopause” for the research topic. For the next stage of selection, only full-text articles were considered for inclusion in further analyses. Up to 30 June 2021, we searched potential articles in the literature from the databases Medline and PubMed. In the screening stage, duplicated articles and articles prior to 1990 were excluded. The literature published from 1 January 1990 to 30 June 2021 was searched to identify eligible articles for the review. Hereafter, two experts in the field independently inspected the contents of articles including basic data, study designs, and research outcomes, and identified potentially eligible studies for inclusion and exclusion. Research articles which had poor study designs or mismatched outcomes were excluded in this stage. To reach a consensus, disagreements between the two experts were discussed by their mutual communication. All eligible articles were included into the topic, using the searching terms and strategies (database searching, article screening, selection of eligible articles, and inclusion). Finally, a total of 108 articles were selected for review.

## 3. Estrogen Deficiency-Related Osteoporosis (EDOP)

### 3.1. Molecular Mechanisms of Actions

**Figure 2:**
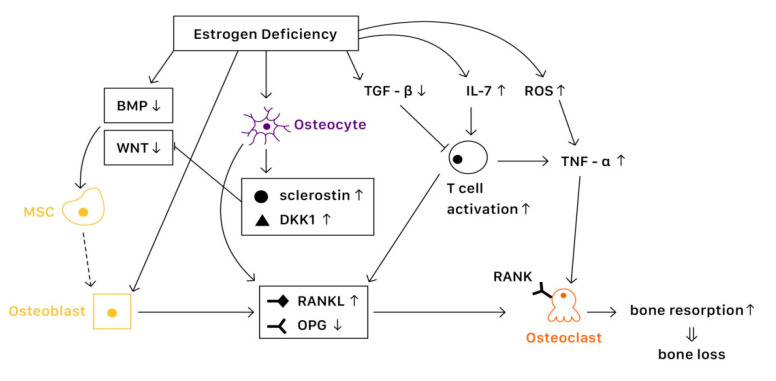
The mechanisms of estrogen deficiency related osteoporosis (EDOP).

It is known that bone remodeling is accomplished by osteoblasts, osteoclasts, and osteocytes. The negative imbalance of bone remodeling, in which bone resorption exceeds bone formation, results in osteoporosis. At a cellular level, several mechanisms contribute to the bone loss related to estrogen deficiency (Figure 2).

#### 3.1.1. Estrogen Signaling

Estrogen receptors (ERs) are highly expressed in osteoblasts, osteoclasts, and osteocytes, offering protective effects in bone. Estrogen binds with ERs, which regulate the expression of estrogen target genes-encoding proteins such as IL-1, insulin-like growth factor 1 (IGF1), and TGFβ [19]. Furthermore, ERs can also suppress the action of nuclear factor-κβ ligand (RANKL), thus inhibiting osteoclast formation and bone resorptive activity [20]. The lack of estrogen will alter the expression of estrogen target genes, increasing the secretion of IL-1, IL-6, and tumor necrosis factor (TNF). Studies have also shown that estrogen deficiency directly affects cell differentiation and apoptosis [21]. The net effects of estrogen deficiency are increased bone turnover and enhanced bone resorption, which result in osteoporosis.

#### 3.1.2. In Osteoblasts

Several pathways are essential to the activation of osteoblast and bone formation, such as canonical WNT, bone morphogenetic protein (BMP), and TGFβ signaling pathway. Almeida et al. demonstrated that ER complex in osteoblast progenitors activated Wnt/β-catenin signaling, thereby increasing osteogenesis [22]. Estrogen is also known to upregulate BMP signaling, which promotes mesenchymal stem cell differentiation from pre-osteoblasts to osteoblasts, rather than adipocytes. Moreover, estrogens stimulate the production of IGF1 and TGFβ by osteoblasts, enhancing bone formation [19].

#### 3.1.3. In Osteoclasts

Various factors are involved in the differentiation and activation of osteoclasts. One key pathway is the NF-κB signaling. The receptor activator of NF-κB (RANK) is expressed on osteoclasts, which is activated when binding with the receptor activator of NF-κB ligand (RANKL) and suppressed when binding with osteoprotegerin (OPG) [23]. Estrogen regulates RANKL and OPG, promoting the expression of OPG and thereby reducing bone resorption. Moreover, estrogen inhibits osteoclast differentiation and advocates osteoclast apoptosis by increasing the production of TGFβ [19]. In the status of estrogen deficiency, RANKL expression is induced, which leads to osteoclastogenesis.

#### 3.1.4. In Osteocytes

Osteocytes serve as mechanosensors and control bone remodeling and mineralization. Lee et al. revealed that in the absence of ERα and its complex, osteocytes were unable to provoke adequate response to mechanical strain, indicating that estrogen deficiency was associated with the impairment of mechanosensors in osteocytes [24]. On the other hand, osteocytes also produce RANKL, which activates osteoclast formation. Additionally, osteocytes inhibit Wnt signaling by forming sclerostin that can bind with Wnt co-receptor LRP5/6, therefore reduce bone formation [23]. In contrast, estrogen retrains the production of sclerostin, protecting bone stability.

#### 3.1.5. Immune Response

Estrogen deficiency leads to the increase of IL-7 to promote the activation of T cells, which induce pro-inflammatory molecules such as IL-1, IL-6, and TNFα, resulting in osteoclast formation [25,26]. Moreover, estrogen deficiency also amplifies T cell activation and osteoclastogenesis by increasing reactive oxygen species (ROS), leading to the production of TNF [26]. Furthermore, RANKL levels are also upregulated in mesenchymal stem cells (MSCs) and T cells as well as in B cells under a lack of estrogen, causing osteoporosis [19].

### 3.2. Risk Factor

#### 3.2.1. Bone Mineral Density

A change in bone mineral density (BMD) is an important predictor for osteoporosis and subsequent fracture. In general, lower BMD is related to a higher risk of fracture. It is estimated that decreasing 1 standard deviation of BMD increases the risk of spine and hip fracture 2.3 to 2.6 fold [27]. Fracture risk can be estimated by Kanis algorithm, which is a model based on BMD, age, and fracture accidents. One cohort study suggested that it can be used in healthy females at menopause. However, underestimation of absolute fracture risks in the Kanis algorithm has been noted [28]. Bone turnover markers are products released during a bone remodeling process, and they correlate with lumbar spine BMD, providing a possible evaluation for the osteoporosis and fracture risks [29].

#### 3.2.2. Genetics

Genetic factors play an important role in osteoporosis. Several single nucleotide polymorphisms (SNPs), such as estrogen receptor α gene (ESR1) and major histocompatibility complex gene (MHC), were associated with age of natural menopause and possible subsequent osteoporosis [30]. Another study revealed that in postmenopausal females, long non-coding RNA (lncRNA) small nucleolar RNA host gene 1 (SNHG1) was down-regulated. An even lower expression level was noted in the postmenopausal osteoporosis group, which might serve as a biomarker for postmenopausal osteoporosis in the future [31].

#### 3.2.3. Menopause Status

Low levels of circulating estrogen are associated with the risk of postmenopausal osteoporosis. Several studies have shown that time since menopause appears to have an association with postmenopausal osteopenia and osteoporosis [32,33,34]. Sioka et al. demonstrated that early menopause between 40 and 45 years of age is correlated with low BMD in postmenopausal females [35]. Svejme et al. found an increased risk of osteoporosis, fragility fractures, and mortality in females with menopause before age 47 [36]. Furthermore, cumulative exposure to estrogen is a protective against osteoporosis. Parker et al. revealed an increased incidence of osteoporosis in females with less than 25 years of menstruation [37]. Due to the fact that premature ovarian insufficiency and early menopause are closely related to osteoporosis, raising concerns and filling up knowledge gaps among the public are needed [38].

#### 3.2.4. Body Weight

Low body weight is a well-documented risk factor for osteoporosis, whereas overweightness is a protective factor [39]. Females with a body mass index (BMI) lower than 20 kg/m^2^ have a significantly higher risk ratio for osteoporosis. On the contrary, for females with a BMI between 25 kg/m^2^ and 35 kg/m^2^, the difference in risk ratio appears smaller [27]. Moreover, another study has suggested that not only body mass, but also the body composition components and segmental distribution of mass in the trunk and leg are important for the prevention of osteoporosis [40].

#### 3.2.5. Lifestyle

Multiple lifestyle factors are associated with a higher osteoporosis risk. Alcohol intake of more than two units daily, cigarette smoking, poor nutrition status, and lack of physical activities are risk factors of osteoporosis. Interestingly, longer sleep durations as well as longer daytime napping times tend to present higher risks of osteoporosis, which could be explained by the reduction of weight-bearing physical activities due to extended sleeping time [41].

#### 3.2.6. Secondary Causes

Various medications and chronic diseases are related to bone loss. There is strong evidence that glucocorticoids are associated with osteoporosis, especially in populations with long term use. In addition, studies have revealed that osteoporosis was found in more than 50% of postmenopausal females and males with rheumatic arthritis. Possible mechanisms for this finding included inflammatory processes and cumulative high glucocorticoid doses [42]. Early menopause due to oncological diseases is also a risk factor of osteoporosis. Oncological treatments, such as surgical, pharmacological, or radiological therapies are associated with iatrogenic menopause and subsequent osteoporosis [43]. Compared with natural menopause, surgical menopause is found to be associated with lower BMD and higher rates of osteoporosis [44,45].

### 3.3. Management

#### 3.3.1. Hormone Therapy

Systemic hormone therapy has shown favorable benefits over harm in females under the age of 60 years or up to 10 years after menopause. For symptomatic postmenopausal females, hormone therapy is a reasonable choice for symptom relief and prevention of bone loss [46]. In females who have undergone hysterectomy, estrogen is given alone. To decrease the risk of endometrial hyperplasia and carcinoma with estrogen use, progestogens and the selective estrogen receptor modulator (SERM) are added into estrogen-base hormone therapy for females with an intact uterus [47]. A novel way of excision of ovarian tissues in the youth and cryostorage for the use after menopause have been discussed. Grafted tissues serve endocrine functions which prevent osteoporosis and menopause-related conditions. Nevertheless, further studies are required for to determine feasibility [48].

#### 3.3.2. Selective Estrogen-Receptor Modulators

Selective estrogen receptor modulators (SERMs) are a class of drugs that can act on estrogen receptors, which act as estrogen agonists or antagonists in different tissues. Raloxifene is the first SERM approved for the treatment of postmenopausal osteoporosis, lowering the risk of vertebral fractures [49]. Bazedoxifene (BZA) is a third-generation SERM with high affinity for the estrogen receptor (ER) alpha. BZA has been combined with conjugated equine estrogen (CEE) to create a tissue selective estrogen complex (TSEC) for the management of vasomotor symptoms (VMS) and the prevention of osteoporosis (OP) associated with menopause [50]. Trials revealed that TSEC has a benefit on improving vulvo-vaginal atrophy, reducing hot flashes and preventing bone loss. There is also a good safety and tolerability profile [51]. TSEC has been approved by the U.S. Food and Drug Administration in 2013 based on five phase 3 studies known as the Selective estrogens, Menopause And Response to Therapy (SMART) trials. For prevention of osteoporosis, the approved dose of BZA/CEE is 20 mg BZA and 0.45 mg CEE [52].

#### 3.3.3. Bisphosphonates

Bisphosphonates, including alendronate, risedronate, and zoledronic acid, are valuable first-line agents of choice in the treatment of postmenopausal osteoporosis [46]. Clinical trials have proven that bisphosphonate increases BMD at hip and vertebral sites significantly in postmenopausal females, reducing fracture risks [27]. A common adverse effect of bisphosphonates is gastrointestinal upset. Despite being widely used, oral bisphosphonates must be administrated with caution in patients with a history of gastroesophageal reflux diseases, Barrett’s esophagus, or peptic ulcers [49].

#### 3.3.4. Calcium and Vitamin D

It is known that many females do not have sufficient calcium and vitamin D intake. Therefore, adequate calcium and vitamin D supplementation is important for building up healthy bone structures in order to prevent osteoporosis. For postmenopausal females, a total daily intake of 1200 mg of calcium and daily supplementation with 800 to 2000 IU of vitamin D are recommended to improve BMD [46].

#### 3.3.5. Parathyroid Hormone (Teriparatide)

Treatment with teriparatide should be considered in postmenopausal females with severe osteoporosis who cannot tolerate other treatments or those who are experiencing new fractures in spite of antiresorptive therapy [46]. Contraindications for teriparatide usage include patients with risks of osteosarcoma, Paget’s disease, hypercalcemia, prior radiation therapy, or a history of previous bone malignancies [49].

#### 3.3.6. RANKL Inhibitor

Denosumab, one of the RANKL inhibitors, is a highly effective and safe treatment for patients with postmenopausal osteoporosis, reducing the risk of vertebral, non-vertebral, and hip fractures [46]. Denosumab is injected every 6 months via a subcutaneous route, which increases compliance, and thus is suggested in patients who cannot tolerate or have failed other treatments. For RANKL inhibitor users, calcium levels should be monitored in patients predisposed to hypocalcemia. Skin infection has been reported as a more commonly noted side effect [27].

#### 3.3.7. Lifestyle Modification

A balanced diet, adequate physical activities, cessation of smoking, and limited alcohol consumption are recommended for osteoporosis prevention [53]. Protein restriction is associated with muscle and bone loss, increasing fragility in bone [54]. An in vivo study demonstrated that olive oil had the property of anti-osteoporosis with an increase in BMD [55]. Therefore, a balanced diet is important for the prevention of osteoporosis. Adequate physical activities have a positive effect on bone health, muscle strength, and balance, which can lower the risk of falling and fractures [56]. In females with fair functional capacity, anaerobic exercise that combines resistance with weight bearing is recommended as well as aerobic exercise. Especially, exercise that focuses on balance should be considered for females at risk of falls [49].

## 4. Glucocorticoid-Induced Osteoporosis (GIOP)

### 4.1. Mechanism

**Figure 3:**
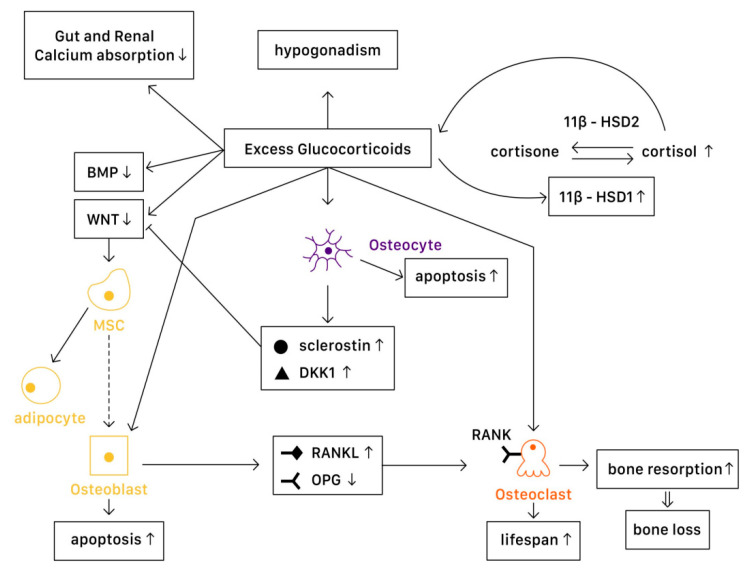
The mechanisms of glucocorticoid-induced osteoporosis (GIOP).

Various levels of glucocorticoids have different effects on osteoblasts, osteoclasts, and osteocytes. Affected by excessive glucocorticoid usage, the most significant activities are the decrease in the proliferation of osteogenic precursors [57] and suppression of osteoblast, reducing bone formation [58]. However, the cellular and molecular mechanisms of glucocorticoids actions in bone remain not fully understood. With advanced technology, studies demonstrate a more detailed understanding on the mechanisms of glucocorticoids on bone turnover (Figure 3).

#### 4.1.1. Physiology

In a physiological state, glucocorticoids stimulate mature osteoblasts to produce Wnt proteins functioning as signaling molecules, causing a Wnt/β-catenin cascade to be activated. This signaling cascade affects mesenchymal progenitor cells, and has a positive effect on their differentiation into osteoblasts instead of chondrocytes or adipocytes. Moreover, Wnt signaling in osteoblasts and osteocytes promotes the production of osteoprotegerin (OPG), a cytokine receptor for NF-κB ligand (RANKL), thus inhibiting osteoclast formation and resulting in decreased bone resorption [7]. Another important pathway is the bone morphogenetic protein (BMP) signaling pathway. Canonical BMP signaling requires the phosphorylation of Smad to activate, which controls the mesenchymal precursor cell’s differentiation, promoting osteoblast differentiation and bone formation [59].

#### 4.1.2. Pathophysiology

##### In Osteoblasts

In a pathophysiological state, excessive glucocorticoids inhibit Wnt protein production, causing mesenchymal progenitor cells to differentiate toward adipocytes rather than osteoblasts. On the other hand, excessive glucocorticoids also interfere with the canonical BMP pathway. One study suggested that in GIOP patients, casein kinase-2 interacting protein-1 (CKIP-1) mRNA and protein expression were both remarkably higher in bone, thus suppressing the Smad-dependent BMP signaling by promoting Smad1 ubiquitination and resulting in the inhibition of osteogenic differentiation [59]. Another study revealed that MiR-106b, a type of miRNA transcribed from the miR-106b-25 cluster, inhibited osteoblastic differentiation of mesenchymal progenitor cells in vitro. In a GIOP mice model, Mi-106b increased its expression and inhibited bone formation partly through targeting BMP2 [60]. Moreover, excess glucocorticoids are associated with osteoblast and osteocyte apoptosis, resulting in declined bone formation [7]. Disruption of canopies that covers the bone remodeling surface was also recognized, leading to insufficient osteoblast recruitment [61].

11β-Hydroxysteroid dehydrogenases (11β-HSDs), two isoenzymes that regulate glucocorticoid activity, also influence the effects of glucocorticoids on bone [7,58]. 11β-HSD type 1 (11β-HSD1) catalyzes the formation of active cortisol, and 11β-HSD type 2 (11β-HSD2) catalyzes the conversion of active glucocorticoids to inactive cortisone. Glucocorticoids are found to enhance 11β-HSD1 expression and activity in osteoblasts, and such enhancement could result in the amplification of the cellular actions of glucocorticoids [58]. Not only in the cellular actions, dysregulation of 11β-HSDs is associated with renal calcium loss in primary male osteoporosis [62], which may also contribute to bone loss. 

##### In Osteoclasts

Excessive glucocorticoids are found to increase RANKL/OPG ratio, which promotes bone resorption by enhancing the maturation and activation of osteoclast. Lekva et al. demonstrated that glucocorticoids increased leucine zipper gene (GILZ) expression, which modulated the expression of OPG [63]. With the reduction of OPG expression, osteoclastogenesis is favored. Additionally, the increase of IL-6 and other cytokines also enhance bone resorption, resulting in bone loss. The lifespan of osteoclast is also prolonged directly by glucocorticoids [7].

##### In Osteocytes

Osteocytes have an important role in bone remodeling, which is dependent on mechanical and hormone signaling. As mentioned above, glucocorticoids are related to osteocyte apoptosis, which reduces bone integrity and increases the risk of fracture. Moreover, excess glucocorticoids induce production of sclerostin and Dickkopf-related protein 1 (DKK1) by osteocytes, thus suppressing the Wnt pathway. Through the activation of PPARγ, mesenchymal progenitors differentiate into adipocytes rather than osteoblasts [7].

##### Systemic Effects 

The mechanisms described above are the direct molecular signaling pathway of glucocorticoid actions. However, when glucocorticoids are given at higher doses, systemic effects cannot be overlooked. The well-documented effects include reductions in calcium absorption in both the gut and the renal tubule that can affect bone mineralization [64]. Hypogonadism in males and premenopausal females is also recognized in high-dose glucocorticoids use, resulting in bone loss [65]. Additionally, avascular necrosis via an apoptotic mechanism of osteocytes and osteoblasts [66] and glucocorticoid-induced myopathy is considered to affect bone health and increase in falling risks [7]. Finally, glucocorticoids alter metabolites and lipid profiles, which potentially have a role in regulating bone loss [67,68].

### 4.2. Assessment and Screening

According to the 2017 ACR guideline, initial clinical fracture risk assessment should be performed at least within 6 months of the initiation of glucocorticoid treatment. For adults over 40 years old, Fracture Risk Assessment Tool (FRAX) with glucocorticoid dose correction and bone mineral density (BMD) testing should be carried out as soon as possible for estimating the absolute fracture risk. As for adults below 40 years old, BMD testing should be carried out if high fracture risks are identified, such as a history of osteoporotic fracture or other significant osteoporotic risk factor [10]. Other than BMD, trabecular bone score (TBS) is a novel analytical tool which can extract data from the two-dimensional lumbar spine dual-energy X-ray absorptiometry (DXA) image. It can provide information relating to trabecular microarchitecture, and is thus capable of greater discriminative power than BMD alone for fracture risk assessment in glucocorticoid-treated patients [69]. For the threshold of therapeutic intervention, individual intervention threshold based on FRAX probability and a higher BMD cut-off point are recommended for GIOP [70,71].

Advanced bone imaging techniques have also been developed, providing structural information beyond BMD with superior discrimination of fracture in GIOP patients [72]. Advanced methods for assessing macrostructure of bone include volumetric Quantitative Computed Tomography (vQCT), peripheral Quantitative Computed Tomography (pQCT), high-resolution Computed Tomography (hrCT), and high-resolution Magnetic Rresonance Imaging (hrMRI) [73]. Although these advanced imaging techniques seem quite promising in the future, several clinical challenges remain, such as cost, radiation exposure, and complexity, which need further exploration. 

### 4.3. Treatment

All adults who are under long-term glucocorticoid therapy should receive adequate intervention on preventing or minimizing GIOP. Unfortunately, a large proportion of patients are neither investigated properly nor treated according to the recommendations [18,74,75]. Nonetheless, pharmacological treatments are at an even lower rate compared with non-pharmacological treatments [76,77].

#### 4.3.1. Non-Pharmacological Treatment

Due to the interference of intestinal calcium absorption and hypercalciuria by glucocorticoids, vitamin D and calcium supplement are recommended [78]. According to the 2017 ACR guideline, all adults taking prednisolone at a dose of over 2.5 mg/day for over 3 months should receive vitamin D (600–800 IU/day) and a calcium supplement (1000–1200 mg/day) [10]. Life modifications are also important for bone health during the treatment.

#### 4.3.2. Pharmacological Treatments

##### Bisphosphonate

Bisphosphonates are the most widely used first-line agents for prevention and treatment in GIOP [79]. Studies have proven that bisphosphonates significantly increase BMD of the lumbar spine, total hip, and femoral neck in patients with GIOP [80,81]. Reducing the risk of vertebral fractures is also identified in patients treated with bisphosphonates in several studies [82,83]. The results of Positron Emission Tomography (PET) scan suggest that bisphosphonate has a direct effect on decreasing bone turnover, resulting in increased BMD [84]. Urinary deoxypyridinoline (DPD), a marker of bone resorption, also decreases after bisphosphonate use [85]. Some common oral forms of bisphosphonate agents include alendronate, etidronate, and risedronate. Injected bisphosphonate medication, such as zoledronic acid, is also available.

For moderate and high risks of major fracture patients, oral bisphosphonate is recommended over calcium and vitamin D alone [10,86]. Oral bisphosphonate (e.g., risedronate) is also recommended as initial treatment over IV bisphosphonate (e.g., zoledronic acid) due to its safety and cost in adults [10]. Although a study of comparing zoledronic acid with risedronate in GIOP males showed significantly greater increases in lumbar spine BMD and total hip BMD in the zoledronic acid group, further safety evaluation is needed for IV bisphosphonate [87]. A combination therapy of plastrum testudinis extract and alendronate in rat spine has shown synergic effects on preventing GIOP; however, more studies should be conducted for further confirmation and application [88].

##### Calcitonin

Calcitonin is a hormonal agent that inhibits bone resorption, with both injected and inhaled forms available. According to previous studies, calcitonin increases BMD in GIOP; however, there is no effect shown on fracture reduction [83,89,90]. Due to its modest efficacy and less effective actions than bisphosphonate, it is not considered as a first-line treatment of GIOP [89,91].

##### Hormone Replacement Therapy (HRT)

Hypogonadism is a side effect of long term corticosteroid use; thus, hormone replacement therapy (HRT) is considered as a second-line or third-line treatment in GIOP patients with sex hormone deficiency [7]. Estrogen has been shown to increase BMD in GIOP patients with sex hormone deficiency [92]; however, increased risks of cardiovascular diseases and breast cancer have significantly reduced its bone benefit. Alternatively, testosterone in males with hypogonadism has been shown to prevent bone loss at the lumbar spine in GIOP [92,93]. On the other hand, the selective estrogen-receptor modulator (SERM) raloxifene also reduces fracture occurrence in postmenopausal females [7,90]. Due to a lack of adequate data and potential harms, raloxifene is only conditionally recommended in postmenopausal females who are not appropriate for any other GIOP medications [10].

##### Teriparatide

Teriparatide is a human recombinant parathyroid hormone (PTH) that increases BMD and reduces the risk of fracture. It activates osteoblasts, which subsequently lead to an increase in bone growth. Teriparatide can also reduce the cellular ROS level elevated by the actions of glucocorticoids to facilitate the proliferation of osteocytes [94].

One randomized controlled trial showed significantly greater increases in spine and hip BMD in the teriparatide group compared with the alendronate group during a 36-months therapy [95]. Increases in biomarkers of bone formation in the teriparatide group with decreases in biomarkers in the alendronate group were also noted [95,96,97]. Along with the bone effects of teriparatide, the decrease in serum HbA1c with some improvement in glucose homeostasis in patients with diabetes and GIOP was recognized among teriparatide users [98]. Despite the fact that many studies have shown teriparatide is more clinically efficacious than bisphosphonates for GIOP, cost burden is still an important issue to be solved [99]. For the time being, bisphosphonate remains the first-line treatment due to its cost effectiveness [10].

##### Denosumab

Denosumab is a RANKL inhibitor that acts similarly as endogenous osteoprotegerin, which reduces bone loss and increases bone density. One randomized, active-controlled trial revealed that denosumab was superior in increasing BMD in lumbar spine and total hip compared with risedronate [100]. The combination therapy of denosumab and teriparatide in a mouse model was shown to increase BMD in the lumbar spine and regenerated cancellous bone volume compared with a single administration of each agent [101]. In spite of the promising future of denosumab in treating GIOP, more safety data in patients under an immunosuppressant status need to be collected in the future.

##### Strontium Ranelate

As an alternative medication for osteoporosis, strontium ranelate consists of strontium salts of ranelic acid, and acts to increase BMD by both enhancing deposition of new bone by osteoblasts and reducing the resorption of bone by osteoclasts. Because the nucleus of strontium is almost the same size as that of calcium, it is easily incorporated into bones to increase bone formation. Furthermore, strontium ranelate promotes the differentiation of pre-osteoblasts to osteoblasts and stimulates osteoblasts to produce osteoprotegerin, which can block the action of RANKL to inhibit the differentiation of preosteoclasts to osteoclasts. In clinical practice, it can significantly lower the risks of vertebral and hip fractures when compared with placebo. However, the serous side effect of strontium ranelate has largely limited its use because of an increased risk of myocardial infarction noted in randomized trials. Currently, the clinical use is restricted to the treatment of severe osteoporosis in postmenopausal females who are at high risk for fractures.

##### Future Possible Treatments

Several experiments targeting on different GIOP pathways were conducted. One study revealed that alpha-lipoic acid, an endogenous anti-oxidant, had a positive effect on improving osteopenia by inhibiting oxidative stress and apoptosis in rat models [102]. Another study targeting on sialic acid-binding immunoglobulin-like lectin 15 (Siglec-15), an immunomoderator that regulated terminal differentiation of osteoclasts, showed promising results on protective effects against glucocorticoid-induced bone loss by suppressing bone resorption in anti-Siglec-15 therapy [103]. On the other hand, herbal medications such as red ginseng, chicory, and curcumin might also have protective effects on GIOP [104,105,106]. However, their effects remain to be investigated due to the small sample size of the existent studies.

## 5. Discussion

Both estrogen deficiency related osteoporosis (EDOP) and glucocorticoid-induced osteoporosis (GIOP) are hormone-related osteoporosis, which account for a large proportion of osteoporosis among the general population. Various mechanisms contribute to the imbalance of bone remodeling including downregulation of BMP and WNT signaling pathways and upregulation of RANKL expression. Activation of T cell and cytokines also plays an important role. The interference in these signaling pathways inhibits the maturation of osteoblasts and osteocytes and increases osteoclastogenesis. Therefore, bone resorption exceeds bone formation, eventually ending up in osteoporosis. Despite the similarity of mechanisms in EDOP and GIOP, studies have shown that intramembranous ossification and endochondral ossification are impaired differently between EDOP and GIOP. In two established bone models, delayed intramembranous ossification is more severe in EDOP compared with GIOP, while endochondral ossification is at the same level [107].

Several common risk factors have been identified among EDOP and GIOP, for instance BMD, age, body weight, and chronic diseases. Other risk factors include early menopause in EDOP and cumulative glucocorticoid dose in GIOP. The most-used screening tools are Fracture Risk Assessment Tool (FRAX) and BMD testing. Early identification of bone loss and early treatment are crucial among populations with a high risk of osteoporosis and fracture. 

The main focuses of treatment for EDOP and GIOP are somewhat different. Avoiding excessive glucocorticoid use is mandatory in patients with GIOP. In contrast, appropriate estrogen supplement is deemed the primary treatment for females with EDOP of various causes including natural menopause, primary and early ovarian insufficiency, or surgical menopause. Non-pharmacological treatments for both EDOP and GIOP include lifestyle modification, vitamin D, and calcium supplements. As for pharmacological treatments, bisphosphonates are the first-line treatment of EDOP and GIOP, which significantly increase BMD and reduce fracture risks. According to the treatment guideline, oral form is prior to intravenous form [10]. Hormone replacement therapy with a combination of estrogens and progestins is the primary therapy in females with EDOP. For those who have undergone hysterectomy, estrogen-only therapy is indicated. SERMs, parathyroid hormone, and RANKL inhibitor are considered as second-line or third-line treatments if the patient failed the initial treatment. It is worth noting that hormone replacement therapy has been used among postmenopausal females for symptomatic relief and osteoporosis prevention as well as treatment of EDOP. However, in GIOP, SERM is only conditionally recommended in postmenopausal females. Due to a better understanding of the pathogenesis of osteoporosis, new treatments have been developed, such as anti-sclerostin, anti-DKK1 treatment, and analog of PTH-related protein [56,108]. The debut of these newer drugs with possible fewer adverse effects and better efficacy is anticipated in the future.

The pathophysiological pathways of osteoporosis as well as its treatment are not fully understood. More studies are still needed to clarify the role of hormone imbalance in osteoporosis. To minimize the heterogeneity of studies in the future, standardization of important factors in research of osteoporosis and its treatment should be considered. One of the critical factors is “time to menopause”, which has an important impact on the individual’s response to estrogen deficiency. Furthermore, durations and outcomes of pharmacological treatments also require standardization, along with the key endocrinal profiles of influenced females. Moreover, a larger sample size is needed to draw a reliable conclusion and improve the replicability and efficacy of the research results.

## 6. Conclusions

Estrogen binds with estrogen receptor to promote the expression of osteoprotegerin (OPG), and to suppress the action of nuclear factor-κβ ligand (RANKL), thus inhibiting osteoclast formation and bone resorptive activity. It can also activate Wnt/β-catenin signaling to increase osteogenesis, and upregulate BMP signaling to promote mesenchymal stem cell differentiation from pre-osteoblasts to osteoblasts, rather than adipocytes. The lack of estrogen will alter the expression of estrogen target genes, increasing the secretion of IL-1, IL-6, and tumor necrosis factor (TNF). On the other hand, excessive glucocorticoids interfere the canonical BMP pathway and inhibit Wnt protein production, causing mesenchymal progenitor cells to differentiate toward adipocytes rather than osteoblasts. It can also increase RANKL/OPG ratio to promote bone resorption by enhancing the maturation and activation of osteoclast. Moreover, excess glucocorticoids are associated with osteoblast and osteocyte apoptosis, resulting in declined bone formation. The main focuses of treatment for EDOP and GIOP are somewhat different. Avoiding excessive glucocorticoid use is mandatory in patients with GIOP. In contrast, appropriate estrogen supplement is deemed the primary treatment for females with EDOP of various causes. Other pharmacological treatments include bisphosphonate, teriparatide, and RANKL inhibitors. Nevertheless, more detailed actions of EDOP and GIOP along with the safety and effectiveness of medications for treating osteoporosis warrant further investigation.
